# Hybrid speciation driven by multilocus introgression of ecological traits

**DOI:** 10.1038/s41586-024-07263-w

**Published:** 2024-04-17

**Authors:** Neil Rosser, Fernando Seixas, Lucie M. Queste, Bruna Cama, Ronald Mori-Pezo, Dmytro Kryvokhyzha, Michaela Nelson, Rachel Waite-Hudson, Matt Goringe, Mauro Costa, Marianne Elias, Clarisse Mendes Eleres de Figueiredo, André Victor Lucci Freitas, Mathieu Joron, Krzysztof Kozak, Gerardo Lamas, Ananda R. P. Martins, W. Owen McMillan, Jonathan Ready, Nicol Rueda-Muñoz, Camilo Salazar, Patricio Salazar, Stefan Schulz, Leila T. Shirai, Karina L. Silva-Brandão, James Mallet, Kanchon K. Dasmahapatra

**Affiliations:** 1 Department of Organismic and Evolutionary Biology, Harvard University, Cambridge, MA USA; 2 Department of Biology, University of York, York, UK; 3 URKU Estudios Amazónicos, Tarapoto, Perú; 4 Universidad Nacional Autónoma de Alto Amazona, Yurimaguas, Perú; 5 Department of Clinical Sciences, Lund University Diabetes Centre, Malmö, Sweden; 6 Residencial Las Cumbres, Caracas, Venezuela; 7 Institut Systématique, Evolution, Biodiversité, UMR 7205 MNHN-CNRS-EPHE-UPMC Sorbonne Universités, Muséum National d’Histoire Naturelle, Paris, France; 8 Smithsonian Tropical Research Institute, Panama City, Panama; 9 Institute for Biological Sciences, Federal University of Pará (UFPA), Belém, Brazil; 10 Centre for Advanced Studies of Biodiversity (CEABIO), Belém, Brazil; 11 Departamento de Biologia Animal and Museu de Diversidade Biológica, Instituto de Biologia, Universidade Estadual de Campinas, São Paulo, Brazil; 12 Centre d’Ecologie Fonctionnelle et Evolutive, UMR 5175 CNRS, Université de Montpellier–Université Paul Valéry Montpellier–EPHE, Montpellier, France; 13 Museo de Historia Natural, Universidad Nacional Mayor de San Marcos, Lima, Peru; 14 Redpath Museum, McGill University, Montreal, Quebec Canada; 15 Biology Program, Faculty of Natural Sciences, Universidad del Rosario, Bogotá, Colombia; 16 Ecology and Evolutionary Biology, School of Biosciences, University of Sheffield, Sheffield, UK; 17 Institut für Organische Chemie, Technische Universität Braunschweig, Braunschweig, Germany; 18 Leibniz Institute for the Analysis of Biodiversity Change, Museum de Natur Hamburg Zoology, Hamburg, Germany; 19 Leverhulme Centre for Anthropocene Biodiversity, Department of Biology, University of York, York, UK

**Keywords:** Speciation, Biodiversity, Evolutionary genetics, Ecological genetics, Quantitative trait loci

## Abstract

Hybridization allows adaptations to be shared among lineages and may trigger the evolution of new species^1,2^. However, convincing examples of homoploid hybrid speciation remain rare because it is challenging to demonstrate that hybridization was crucial in generating reproductive isolation^3^. Here we combine population genomic analysis with quantitative trait locus mapping of species-specific traits to examine a case of hybrid speciation in *Heliconius* butterflies. We show that *Heliconius elevatus* is a hybrid species that is sympatric with both parents and has persisted as an independently evolving lineage for at least 180,000 years. This is despite pervasive and ongoing gene flow with one parent, *Heliconius pardalinus*, which homogenizes 99% of their genomes. The remaining 1% introgressed from the other parent, *Heliconius melpomene*, and is scattered widely across the *H. elevatus* genome in islands of divergence from *H. pardalinus*. These islands contain multiple traits that are under disruptive selection, including colour pattern, wing shape, host plant preference, sex pheromones and mate choice. Collectively, these traits place *H. elevatus* on its own adaptive peak and permit coexistence with both parents. Our results show that speciation was driven by introgression of ecological traits, and that speciation with gene flow is possible with a multilocus genetic architecture.

## Abstract (web summary)

Genomic studies of *Heliconius* butterflies provide evidence that *Heliconius elevatus* is a hybrid species, and that its speciation was driven by introgression of traits from *Heliconius melpomene* into the other parent, an ancestor of *Heliconius pardalinus*.

## Main

**Fig. 1:**
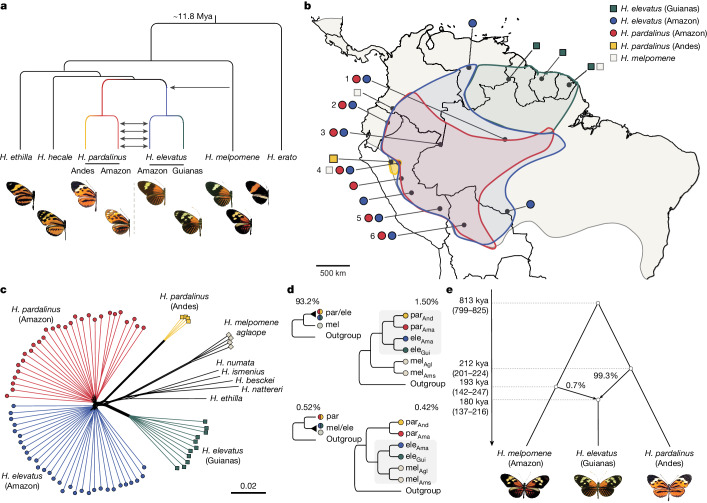
Summary of key findings, geographical distributions of species and evidence that *H. elevatus* has a hybrid genome. **a**, Evolutionary relationships and main introgression events described in this study. We test the hypothesis that introgression of major pre-mating and post-mating ecological isolating traits from *H. melpomene* led to the establishment of *H. elevatus* as a new stable hybrid species. Mya, million years ago. **b**, Geographical distributions of major clades. Locations at which both *H. elevatus* and *H. pardalinus* were sampled are numbered. **c**, Distance-based network using genome-wide independent SNPs. This concatenated tree shows the existence of two distinct clusters, Amazonian versus non-Amazonian, in both *H. elevatus* and *H. pardalinus*. **d**, Topology weighting analysis (TWISST) showing the percentage of the 11,509 non-overlapping genomic windows of 1,000 SNPs in which the majority of subtrees (that is, topology weighting ≥ 0.5) clusters *H. elevatus* (ele) with either *H. pardalinus* (par) (93.2%; top) or *H. melpomene* (mel) (0.52%; bottom). Note that although *H. elevatus* groups with *H. pardalinus* in 93.2% of windows, only 1.61% of those trees yield the two species as reciprocally monophyletic. By contrast, all three species are monophyletic in 81% of the windows for which *H. elevatus* groups with *H. melpomene*. Subscripts indicate geographical distributions for *H. elevatus* and *H. pardalinus* (Ama, Amazon; And, Andes; Gui, Guianas) and subspecies for *H. melpomene* (Agl, *aglaope*; Ams, *amaryllis*). **e**, A multispecies coalescent model with introgression supports a hybrid origin of *H. elevatus*, with the introgression time coinciding closely with the origin of the species (the 95% HPD intervals are given within parenthesis). Images of butterfly wings are copyright of the authors and Michel Cast.

Biodiversity has long been depicted as a ‘tree of life’, but a wealth of genomic data has made clear that the branches and leaves of the tree often do not represent neatly defined units. Instead, they comprise a braided delta of evolutionary lineages linked by hybridization and introgression^4^. Although gene flow tends to homogenize populations^5^, it may also contribute to adaptation and even drive speciation if introgressed variants cause reproductive isolation^1,2,6^. Polyploid (chromosome-doubling) hybrid speciation is common in plants^7,8^, but compelling examples of homoploid hybrid speciation (without a change in chromosome number) remain scarce and contested, especially in animals^3^. This is because it is difficult to prove that hybridization had a pivotal role in creating reproductive isolation between the hybrid lineage and the parental species^3^. Here we present evidence for homoploid hybrid speciation in *Heliconius* butterflies. We show that introgression of key adaptive traits from *H. melpomene* caused *H. elevatus* to diverge from *H. pardalinus*, despite ongoing gene flow among sympatric *H. elevatus* and *H. pardalinus*, which homogenizes 99% of their genomes (Fig. 1a).

*Heliconius* butterflies are chemically defended by cyanogenic glycosides, either sequestered from larval passion-vine host plants (Passifloraceae) or synthesized de novo^9,10^. Adults signal their toxicity to predators through their brightly coloured wing patterns. The cost of educating predators is shared with other defended butterflies and moths through mutualistic Müllerian mimicry^11^. Mimicry among species is not restricted to colour pattern, because co-mimics also converge in flight behaviour and wing shape^12,13^. Hybrids with intermediate phenotypes are selected against because predators do not recognize them^14,15^. Therefore, different mimetic phenotypes correspond to fitness peaks in an adaptive landscape maintained by disruptive selection. How populations transition to new fitness peaks remains an unanswered question, but adaptive introgression provides a possible route.

*Heliconius elevatus*, *H. pardalinus* and *H. melpomene* present an excellent system with which to elucidate the role of hybridization in speciation. All three species are sympatric across the Amazon basin^16–18^ (Fig. 1b). *Heliconius elevatus* and *H. pardalinus* are closely related^19^, but they have strikingly different colour patterns (Fig. 1a). *Heliconius pardalinus* has a ‘tiger’ mimetic colour pattern typical of its close relatives. By contrast, *H. elevatus* has a red, black and yellow pattern, mimicking the much more distantly related *H. melpomene*^19^. This phenotypic convergence results in part from introgression at the major colour patterning loci *cortex* and *optix*^20^. Because *Heliconius elevatus* uses colour pattern as a partial cue in mate choice^17^, these introgressed alleles are likely to promote pleiotropic reproductive isolation from *H. pardalinus*. This suggests that *H. elevatus* has a hybrid origin, and here we test that hypothesis.

## Hybrid genome of *H. elevatus*

**Extended Data Fig. 1:**
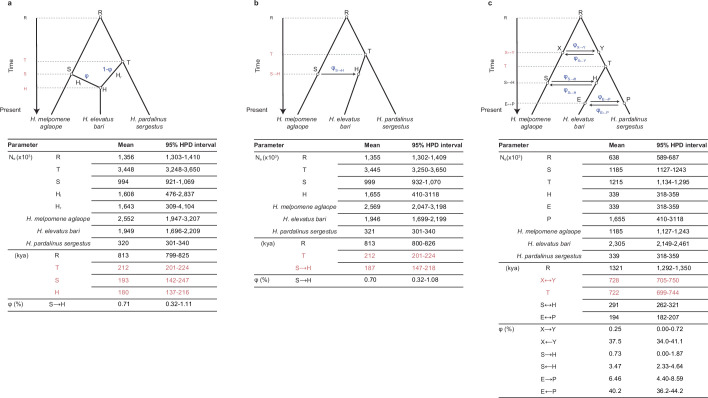
Evaluating the hybrid speciation hypothesis under the MSCi model. For each model, a schematic representation is depicted on top and the estimated values under the MSCi are presented in the table below. In the schematics, open circles denote internal nodes and arrows between internal nodes represent single migration pulses. Effective population sizes (N_e_) are scaled to thousand individuals. The age (*τ*) of splits and nodes involved in hybridization events are given in kya. Introgression probabilities (*φ*) are depicted in blue and are given as a percentage. **a**, Model in which the two parental species, S (ancestral of *H. melpomene*) and T (ancestral of *H. pardalinus*), hybridize to originate the hybrid lineage H (ancestral of *H. elevatus*), with *φ* contribution from parent S and 1-*φ* from parent T. The nodes S and T may have distinct ages and are older than node H. **b**, Same as model **a**, but constraining introgression from S into H to occur after an initial split between *H. elevatus* and *H. pardalinus* (*τ*_H_ *<* *τ*_T_). Introgression is instantaneous and occurs at time *τ*_H_ (which is the same as *τ*_S_). **c**, Model allowing bidirectional migration between an *H. melpomene* ancestor (X) into the ancestral population of *H. elevatus* and *H. pardalinus* (Y), between an *H. melpomene* ancestor (S) and the lineage leading to *H. elevatus* (H), and between the lineage leading to *H. elevatus* (E) and the lineage leading to *H. pardalinus* (P). Note that in all models, the 95% HPD intervals of the age of gene flow from *H. melpomene* into *H. elevatus* and the split between *H. elevatus* with *H. pardalinus* overlap, in line with *H. elevatus* being a hybrid lineage. These are highlighted in red.

We analysed whole-genome sequences of 92 wild-caught individuals of these three species: 42 *H. elevatus* (12 locations), 33 *H. pardalinus* (7 locations) and 17 *H. melpomene* (4 locations). For *H. elevatus* and *H. pardalinus*, our sampling spanned their combined geographical range (Fig. 1b and Supplementary Table 1). A concatenated whole-genome phylogenetic network groups *H. pardalinus* with *H. elevatus*, whereas *H. melpomene* forms a much deeper lineage separated by several other species^21^ (Fig. 1c). This topology is echoed in 93.2% of the genealogies estimated in sliding windows of 1,000 single-nucleotide polymorphisms (SNPs) across the genome, whereas 0.52% of the genealogies cluster *H. elevatus* with *H. melpomene* (Fig. 1d). This is suggestive of introgression between these two species but could also be explained by retention of ancestral polymorphisms. Testing this hypothesis under the multispecies coalescent with introgression framework^22^ (Fig. 1e), we find that most of the *H. elevatus* genome is derived from *H. pardalinus*, with a 0.71% (95% high posterior density (HPD) 0.32–1.11%) contribution from *H. melpomene*. *Heliconius elevatus* arose as an independent lineage around 180,000 years ago (kya) (Fig. 1e; 95% HPD 137–216 kya; see also Extended Data Fig. 1). This divergence time coincides closely with the divergence from *H. pardalinus* (212 kya, 95% HPD 201–224 kya) and the timing of introgression from *H. melpomene* (193 kya, 95% HPD 142–247 kya). These coalescent-based estimates are therefore consistent with *H. elevatus* being a hybrid lineage that formed through admixture between *H. pardalinus* and *H. melpomene*.

## Ongoing local gene flow with *H. pardalinus*

**Extended Data Fig. 2:**
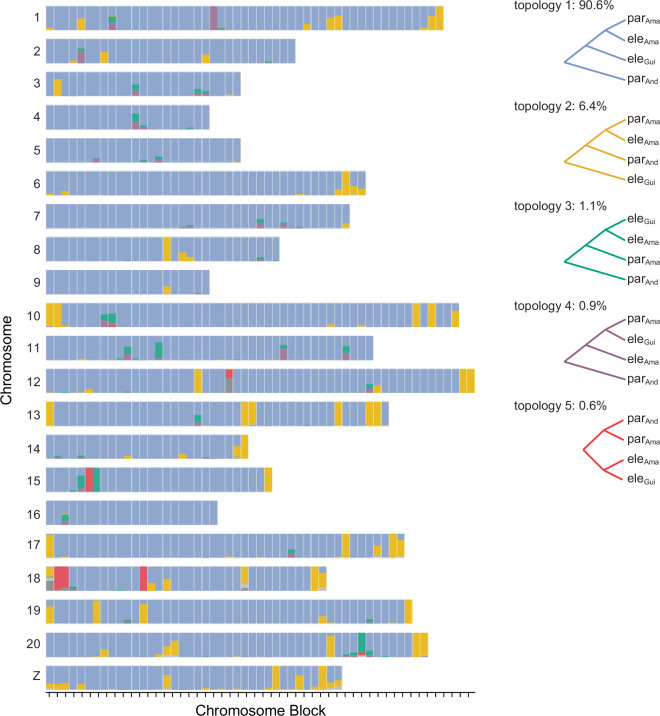
MSC analysis of *H. elevatus* and *H. pardalinus*. Species-tree phylogeny along chromosomes calculated in blocks of 100 loci using BPP (ref. ^22^). Only the five major topologies are depicted (minor topologies are coloured in grey).

Notably, the whole-genome concatenated phylogenetic network suggests that sympatric populations of *H. elevatus* and *H. pardalinus* in the Amazon are more closely related to each other than to allopatric conspecifics from the Peruvian Andes and the Guianas (Fig. 1c). Only 1.92% (1.50% + 0.42%; Fig. 1d) of the 11,509 windows across the genome yield reciprocally monophyletic genealogies for *H. pardalinus* and *H. elevatus*; this is confirmed by multispecies coalescent-based trees across the genome (Extended Data Fig. 2). We therefore investigated whether this apparent double species paraphyly could be explained by extensive ongoing gene flow between *H. elevatus* and *H. pardalinus* in sympatry in the Amazon.

**Fig. 2:**
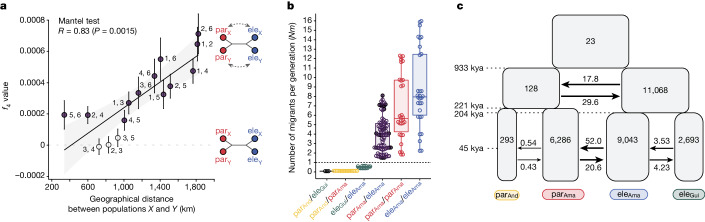
Genomic admixture between *H. elevatus* and *H. pardalinus*. **a**, Gene flow between species in sympatry is typically significantly greater (*f*_4_ > 0, filled points) than the within-species gene flow between populations across the Amazon basin. Numbers next to the points indicate the population pairs compared (see Fig. 1b). A significant positive correlation indicates that the within-species gene flow between populations *X* and *Y* declines with increasing geographical distance relative to between-species gene flow at populations *X* and/or *Y*. **b**, Estimates of effective migration rate (*Nm*) within and between species using G-PhoCS. In the Amazon, between-species *Nm* (par_Ama_/ele_Ama_) is similar to within-species *Nm* between locations (par_Ama_/par_Ama_ and ele_Ama_/ele_Ama_). The estimates for par_Ama_/ele_Ama_ are denoted as filled and open circles and correspond to within and between location comparisons, respectively. **c**, The best supported demographic model based on the site-frequency spectrum analysis supports *H. elevatus* and *H. pardalinus* as reciprocally monophyletic (Extended Data Fig. 4). They initially diverged with continuous gene flow (933 to 221 kya) and after splitting into Amazonian and non-Amazonian populations, they came into secondary contact and continued exchanging genes until the present (45 kya to the present). Numbers within the blocks are effective population sizes in thousands. Arrows between groups represent continuous gene flow; numbers above or below arrows indicate 2*Nm* values.

**Extended Data Fig. 3:**
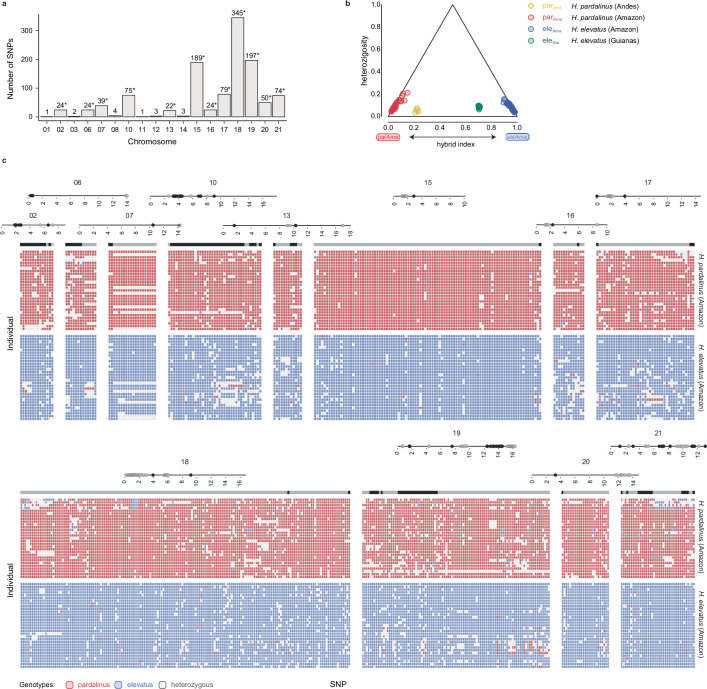
Species-diagnostic SNPs. **a**, Number of species-diagnostic SNPs per chromosome. Species-diagnostic SNPs were defined as SNPs with an allelic difference of at least 0.8 between all Amazonian populations of *H. elevatus* and *H. pardalinus*. Chromosomes with at least 20 diagnostic SNPs are denoted with an asterisk (*) and shown in more detail in **c**. **b**, Triangular plot of hybrid index and observed heterozygosity, based on the 1,156 species-diagnostic SNPs, shows no evidence of early generation hybrids. **c**, Distribution of species-diagnostic SNPs along chromosomes in wild-caught *H. elevatus* and *H. pardalinus*. The physical location of SNPs along chromosomes (in Mb) are shown on top. Different blocks of SNPs within a chromosome, defined as groups of SNPs more than 500 kb apart, are denoted in alternating colours (black and grey). For visualization purposes, only chromosomes with at least 20 diagnostic SNPs are shown and SNP blocks were subsampled to show only one in every two SNPs. Long tracts of heterozygous genotypes (e.g. chromosome 19) suggest relatively recent hybridization followed by backcrossing.

**Extended Data Fig. 4:**
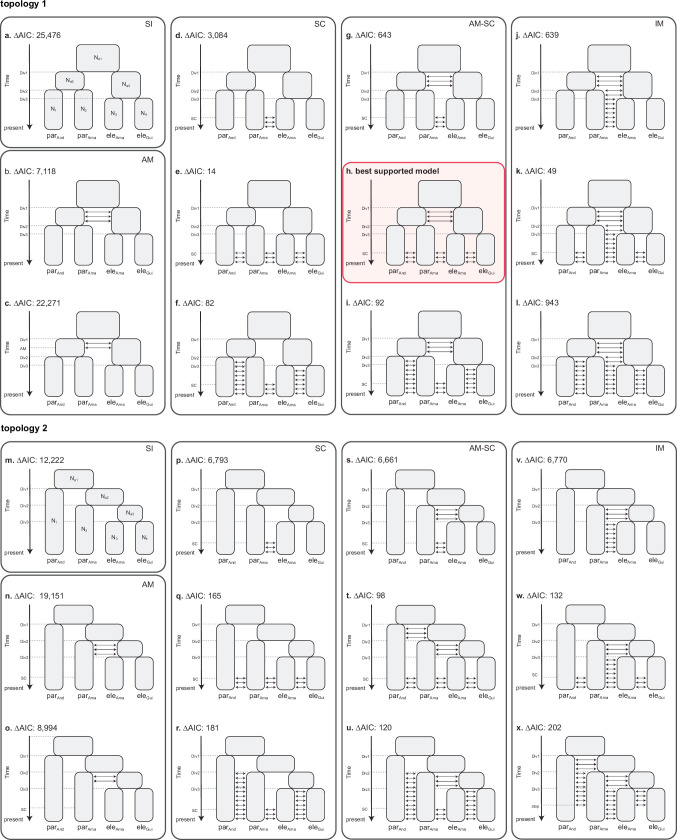
Schematic of all demographic models tested with fastsimcoal2. Two different tree topologies and 12 models per topology were tested. We considered the topology that retrieves both *H. elevatus* and *H. pardalinus* as monophyletic; that is, the species tree, (topology 1) and the most frequent topology across the genome (Extended Data Fig. 2), after excluding gene flow between *H. elevatus* and Amazonian *H. pardalinus* and in which *H. p. sergestus* is the first population to split (topology 2). The different demographic models are split into five main categories (depicted in different boxes): SI, strict isolation; AM, ancestral migration; SC, secondary contact; AM-SC, ancestral migration followed by secondary contact; IM, isolation with migration. Arrows between demes indicate gene flow (each direction being estimated as an independent parameter). Effective population sizes were allowed to change at split times. Note that for models under tree topology 1, the split times between *H. elevatus* populations and between *H. pardalinus* populations are different parameters and thus can assume different values.

Putative natural hybrids have been reported occasionally between *H. elevatus* and *H. pardalinus*^23^, but the two very rarely mate in captivity^17^. However, F_1_ hybrids from forced matings are fully fertile^17^. We therefore examined the genomes of wild-caught individuals of the two species from sympatric populations across their range for evidence of gene flow. Focusing on SNPs diagnostic for *H. elevatus* and *H. pardalinus*, we find a few individuals with long tracts of heterozygous ancestry, in some cases spanning whole chromosomes, indicating recent gene flow (Extended Data Fig. 3). Four-population (*f*_4_) tests comparing within- and between-species gene flow support ongoing interspecific gene flow in sympatry (Fig. 2a). Estimated levels of effective gene flow between the species in sympatry are high (*Nm* > 1, where *N* is the effective population size, and *m* is the migration rate per generation), quite sufficient to homogenize neutral variation between species; indeed, gene flow approaches the rates that are found among nearby populations of the same species (Fig. 2b and Supplementary Table 2). Finally, we performed demographic modelling based on the site-frequency spectrum under different demographic scenarios and topologies. The best supported model retrieved a tree in which *H. elevatus* and *H. pardalinus* were reciprocally monophyletic, and confirmed that gene flow has been prevalent throughout their combined history (Fig. 2c and Extended Data Fig. 4). After their initial split, populations of *H. pardalinus* in the Amazon diverged from those in the Andes, and Amazonian populations of *H. elevatus* diverged from those in the Guianas (Fig. 2c and Supplementary Table 3). The two species then began to overlap broadly in the Amazon from around 28 kya (95% confidence interval 25.6–30.0 kya) until the present, with high levels of gene flow in sympatry. Nonetheless, sympatric populations of *H. elevatus* and *H. pardalinus* in the Amazon form mutually monophyletic genetic clusters (Fig. 1c); thus, the two species remain differentiated and can clearly coexist despite extensive ongoing gene flow, implying the existence of strong sexual and ecological isolation.

## Lack of gene flow with *H. melpomene*

**Extended Data Fig. 5:**
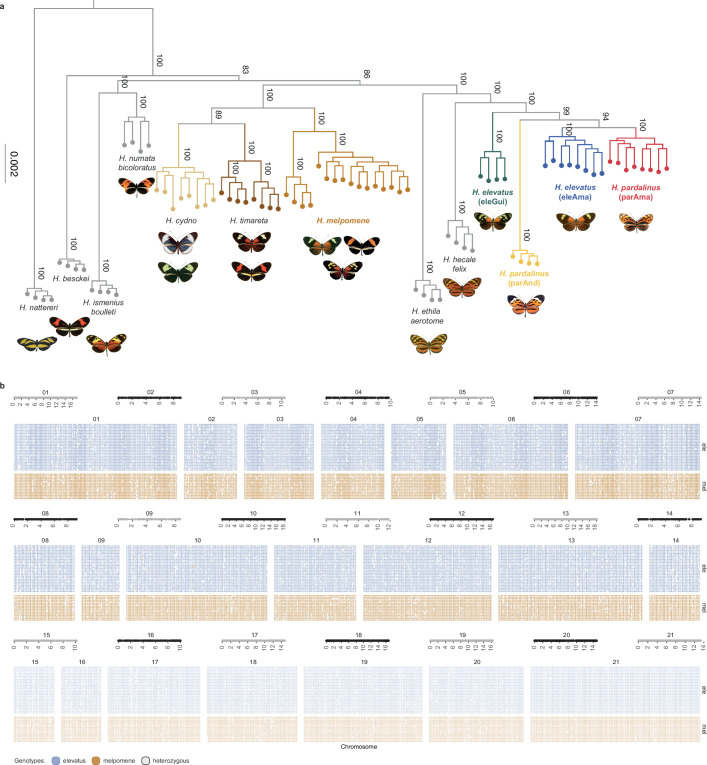
Genetic evidence for current reproductive isolation between *H. elevatus* and *H. melpomene*. **a**, Neighbour-joining tree based on autosomal sites sampled every 1 kb (166,989 sites). Values next to branches denote bootstrap values (based on 100 bootstrap iterations). Images of butterfly wings are copyright of the authors and Michel Cast. **b**, Distribution of species-diagnostic SNPs along chromosomes in wild-caught *H. elevatus* and *H. melpomene*. Species-diagnostic SNPs were defined as SNPs with an allelic difference of at least 0.8 between all Amazonian populations of *H. elevatus* and all *H. melpomene* populations. The physical location of SNPs along chromosomes (in Mb) are shown on top. For visualization purposes, SNP blocks were subsampled to show only 1 in every 20 SNPs. The lack of long tracts of heterozygous genotypes (or introgressed homozygous genotypes) suggests that there is no recent hybridization, followed by backcrossing, between these two species.

**Extended Data Fig. 6:**
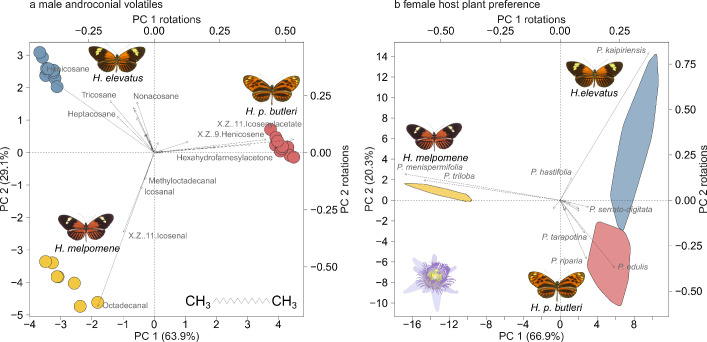
PCAs of male sex pheromones and host plant use show that *H. elevatus*, *H. pardalinus* and *H. melpomene* from the western Amazon form three distinct clusters in trait space. **a**, PCA applied to concentrations of 30 male androconial volatiles. Loadings for selected compounds are annotated. **b**, PCA applied to oviposition preference of *H. elevatus*, *H. pardalinus* and *H. melpomene* for 21 species of *Passiflora*. *Heliconius melpomene* (24 females) laid 288 eggs and exhibited a strong preference for *P. menispermifolia* and *P. triloba*. *Heliconius elevatus* (35 females) laid 173 eggs and exhibited a preference for *P. kaipiriensis*. *H. pardalinus butleri* (51 females) laid 425 eggs and had a more generalized host plant use. To estimate the sample variance for each species, subsamples of 30 were drawn with replacement from the distribution of each species (1,000 replicates). PCA was then run on these bootstrapped replicates, polygons are minimum convex hulls encompassing all subsamples for each species. Images of butterfly wings are copyright of the authors and Michel Cast.

The genome of *Heliconius elevatus* is, on average, more distantly related to its other parental species *H. melpomene* than it is to *H. pardalinus* (Fig. 1d and Extended Data Fig. 5a). Yet gene flow from *H. melpomene* is plausible, because the latter is known to hybridize occasionally with other equally distant species in the wild^24,25^. None of the 31 *H. elevatus* or 17 *H. melpomene* individuals collected from areas of sympatry show any tracts of heterospecific ancestry (Extended Data Fig. 5b). Likewise, *f*_4_ tests do not detect any signals of gene flow (Supplementary Table 4). These data indicate that, in contrast to the extensive ongoing gene flow detected between *H. elevatus* and *H. pardalinus*, any recent gene flow between *H. elevatus* and *H. melpomene* is extremely rare. Because the *H. elevatus* and *H. melpomene* colour pattern phenotypes are essentially identical, this trait is probably not used to discriminate conspecifics^26^. Instead, their coexistence is likely to be due to strong assortative mating mediated by traits such as male sex pheromones and host plants (Extended Data Fig. 6), as well as female-limited hybrid sterility, which evolves rapidly^27,28^ and helps to isolate *H. melpomene* from other sympatric, co-mimetic species^29^.

## Barriers inherited from *H. melpomene*

**Fig. 3:**
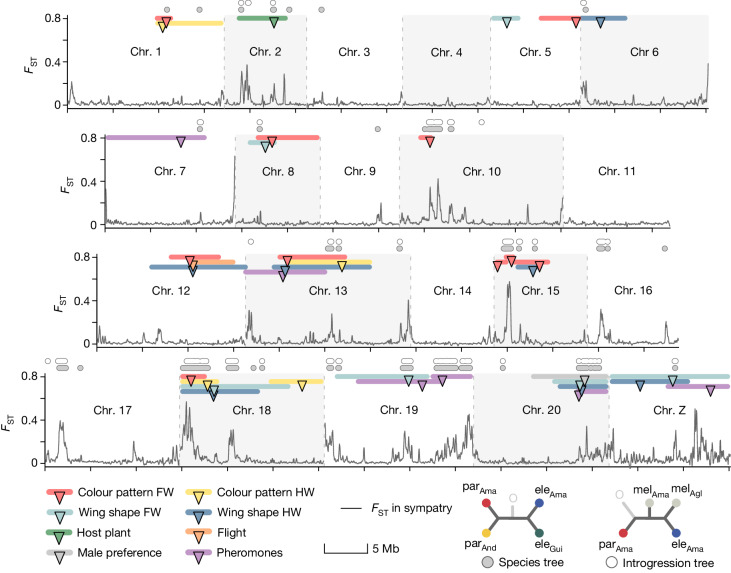
Speciation of *H. elevatus* was driven by multilocus introgression of ecological traits. Patterns of genomic divergence between sympatric *H. elevatus* and *H. pardalinus* in the Amazon together with locations of mapped traits. The black line and *y* axis show *F*_ST_ in 25-kb sliding windows across the genome. Coloured bars show significant QTLs for different traits, with the QTL peak indicated by the triangle and the Bayesian credible intervals by the length of the bar. For colour pattern and wing shape, only QTLs with non-overlapping credible intervals are shown. Most of the genome shows very low *F*_ST_ due to gene flow in the Amazon, causing the double paraphyly topology for *H. elevatus* and *H. pardalinus* in Fig. 1c. Genomic regions with rare phylogenetic topologies (bottom right) supporting introgression from *H. melpomene* (white circles, introgression tree) and resolving the *pardalinus*–*elevatus* species tree (grey circles, species tree) are shown above the plot. These topologies often coincide with one another and with *F*_ST_ peaks. O, outgroup (*Heliconius ethilla*). FW, forewing; HW, hindwing.

As a result of extensive ongoing gene flow, differentiation (*F*_ST_) between sympatric populations of *H. elevatus* and *H. pardalinus* is approximately zero across around 99% of their genomes (Fig. 3). Only around 1% of the genome shows increased differentiation (*F*_ST_ ≥ 0.2) and retrieves both species as reciprocally monophyletic on the basis of topology weighting by iterative sampling of subtrees (TWISST) analysis, comprising 44 genomic islands of divergence. Notably, genealogies within genomic islands resolve all populations of both species, including the peripheral allopatric lineages, as reciprocally monophyletic (Fig. 3). Furthermore, introgression from *H. melpomene* is especially prevalent in these islands and is found in 32 of the 44 genomic islands of divergence (Fisher’s exact test *P* < 0.001; Fig. 3). Because these genomic islands resist homogenization despite gene flow in sympatry, we hypothesize that they contain the genetic basis for species differences.

**Fig. 4:**
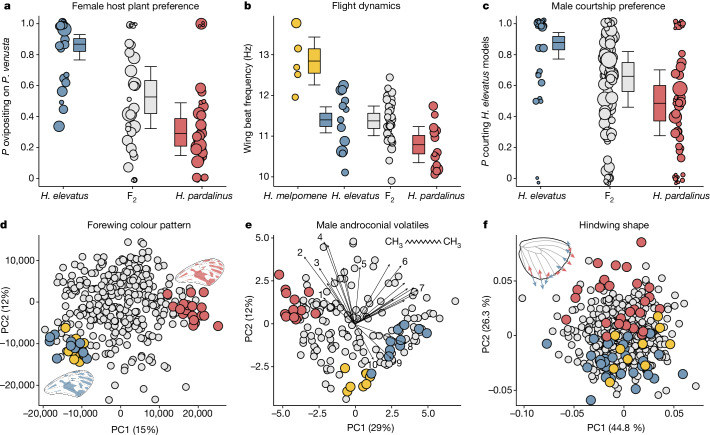
Key species traits under divergent ecological and sexual selection. **a**, *Heliconius elevatus* and *H. pardalinus* differ in host plant preference during egg-laying; female *H. elevatus* show a stronger preference for *Passiflora venusta* relative to *Passiflora riparia*. *Heliconius melpomene* has a very distinct host plant preference and lays eggs on neither of these plants (Extended Data Fig. 6). Point sizes here and in **b**,**c** are scaled by the log of sample size. Error bars are 95% confidence limits. **b**, The two species differ in flight dynamics; *H. elevatus* beats its wings significantly faster than *H. pardalinus*, and converges towards *H. melpomene aglaope*. **c**, Given a choice, male *H. elevatus* individuals preferentially court model female wings with their own colour pattern relative to *H. pardalinus*, whereas *H. pardalinus* males exhibit no preference. **d**, Principal component analysis (PCA) of forewing colour pattern in hybrid crosses with the parental species and *H. melpomene aglaope* rotated and projected into this space. Wings show the top 10% of pixels contributing to the variance in PC1. **e**, PCA of male sex pheromones in hybrid crosses, with parental species rotated and projected. Differences between the species are driven mainly by variance in alkanes. Selected loadings: (1) hexahydrofarnesylacetone; (2) (*Z*)-9-heneicosene; (3) (Z)-11-eicosenylacetate; (4) (*Z*)-9−tricosene; (5) 11-methylhexacosane; (6) 11-methylpentacosane; (7) heptacosane; (8) tricosane; (9) heneicosane (inset figure); and (10) homovanillyl alcohol. **f**, PCA of hindwing shape in hybrid crosses, with parental species rotated and projected. Inset wing shows changes in hindwing shape observed along PC2. Only landmarks along the margin of the wing are shown. Individual specimens are depicted as circles: *H. elevatus*, blue; *H. pardalinus*, red; F_2_s and backcrosses, grey; and *H. melpomene*, yellow.

To understand the genetic architecture of traits that allow the coexistence of *H. elevatus* and *H. pardalinus*, we crossed sympatric Amazonian populations of these species. We identified quantitative trait loci (QTLs) for several species-specific traits, including colour pattern, male sex pheromones, male preference for female colour pattern, wing shape, flight and female host plant preference, in F_2_ and backcross offspring (Fig. 4). These traits contribute to reproductive isolation because they are under divergent selection and/or directly determine mate choice. For example, host preference is likely to be under divergent ecological selection and also confers non-random mating because *Heliconius* mate in the vicinity of their host plants^30,31^. In total, we identified 63 QTLs associated with species differences at these traits, which mapped to 14 of the 21 chromosomes (Fig. 3 and Supplementary Table 5).

QTLs for colour pattern mapped to chromosomes 1, 5, 10, 12, 13, 15 and 18, with those on chromosomes 10, 15 and 18 containing the known colour patterning genes *WntA*, *cortex* and *optix* (refs. ^16–18^). We identified a large effect locus on chromosome 20 that determined variation in hindwing shape (*H. elevatus* ancestry is associated with wider and shorter hindwings). Hybrid flight dynamics were quantified using high-frame-rate video footage. A single locus on chromosome 12 predicted wing beat frequency and explained 43% of the variance. Consistent with species differences (Fig. 4b), individuals with genotype EE (homozygous ancestry for *H. elevatus*) beat their wings faster (11.2 ± 0.1 Hz) than did PP (homozygous ancestry for *H. pardalinus*) individuals (10.9 ± 0.2 Hz), in which E is the *H. elevatus* allele and P is the *H. pardalinus* allele. In controlled insectary experiments, *Heliconius elevatus* females exhibited a strong preference for *Passiflora venusta* relative to *Passiflora riparia* (Fig. 4a), concordant with wild host plant records^17^. A single locus on chromosome 2 predicted the preference of female hybrids for different host plants (Fig. 3); the probability of ovipositing on *P. venusta* increased from 0.3 (s.e. 0.19–0.42) for genotype PP to 0.87 (s.e. 0.81–0.91) for genotype EE.

Mate choice among sympatric populations is further mediated by female preference for male sex pheromones secreted on wing androconia and male preference for colour pattern (attractiveness of females to males)^13^. We found large effect QTLs for male androconial volatiles on chromosomes 19 and 20. These genomic regions (see Supplementary Table 5) contain many genes encoding enzymes that are involved in fatty acid metabolism, such as reductases and Δ9-desaturases^32^—strong candidates for controlling differences between the saturated-fatty-acid-derived androconial volatiles of *H. elevatus*, and the unsaturated-fatty-acid-derived blend of *H. pardalinus*. For example, genotype EE at the chromosome 19 locus is associated with an approximately 100-fold increase in the concentration of heneicosane, relative to genotype PP, with the QTL explaining about a third of the variance.

The male colour pattern preference QTL with the highest LOD score (3.14) was tightly linked to QTLs for androconial volatiles and wing shape on chromosome 20. However, this QTL was not statistically significant. This might be explained if male preference is highly polygenic. In support of this, we found that the probability of a male courting the *H. elevatus* colour pattern is positively correlated with the total fraction of the male’s *H. elevatus* chromosomal ancestry (*P* < 0.05, generalized linear mixed model with binomial errors and individual-level random effect). For comparison, we applied the same test to host plant choice and found no such association, suggesting that host preference has a simpler genetic basis.

Consistent with hybridization driving speciation, QTLs underpinning species-specific traits are linked to genomic windows introgressed from *H. melpomene* far more often than when the position of these QTLs is randomized across the genome (mean recombination rate between QTLs and nearest introgression topology, *c* = 0.26; randomized mean *c* = 0.39; *P* < 0.001). QTL peaks that are tightly linked to *H. melpomene* introgression regions (*c* < 0.05) include those that determine colour pattern mimicry on chromosomes 10, 15 and 18, wing shape on chromosomes 19 and 20, male sex pheromones on chromosome 19 and 20, host plant preference on chromosome 2 and male preference on chromosome 20. Moreover, for colour pattern, wing shape, male sex pheromones and flight behaviour, *H. elevatus* exhibits trait values similar to those of *H. melpomene* (Fig. 4), providing a direct link between introgression, genotype and phenotype. Hence, these loci influencing ecological traits and derived from introgression represent key genomic regions that enabled hybrid speciation (Fig. 3).

**Extended Data Fig. 7:**
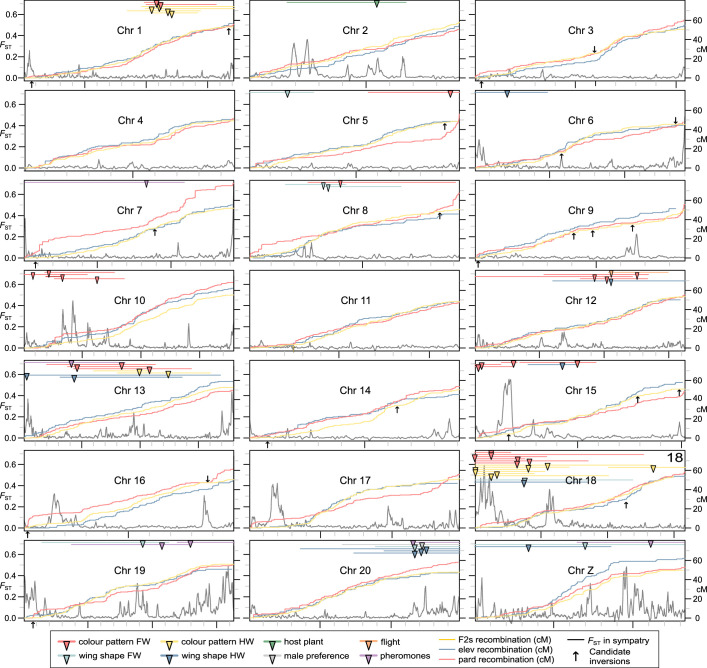
*F*_ST_ and genetic distances plotted against physical distance. Physical distance is shown on the *x* axis; grey intervals are 1 Mb and black intervals are 5 Mb. Coloured bars show significant QTLs, with the QTL peak indicated by the triangle and the Bayesian credible intervals indicated by the length of the bar. Genetic distances are estimated using three crosses—within population (*Heliconius elevatus*; elev and *Heliconius pardalinus*; pard) and between population (F_2_). Candidate inversions (CIs; indicated by black arrows) are regions that recombine within species but not in hybrids (see Methods). The largest CI we identified was around 1.4 Mb long at the distal end of chromosome 16. However, we identified no CIs greater than 870 kb within the credible intervals of QTLs, and only one instance of a CI that was coincident with a QTL peak (on chromosome 15). Nonetheless, some CIs outside of QTLs present compelling targets for future investigation. Notably, at the proximal end of chromosome 19 and the distal end of chromosome 16, two large CIs overlap regions with elevated *F*_ST_ in which phylogenies resolve species boundaries (see Fig. 3).

Linkage or pleiotropy among traits are often thought to be necessary to circumvent the homogenizing effect of gene flow^5,33^. After removing overlapping loci in the colour pattern and wing shape phenotypic classes (Fig. 3), 28% of QTLs were tightly linked to at least one other species trait locus (recombination fraction *c* < 0.05), and only 11% were completely unlinked (*c* ≈ 0.5). The mean recombination fraction (*c*) between trait loci and their nearest neighbour was significantly lower than when positions of the loci were randomized across the genome (observed mean *c* = 0.26; randomized mean *c* = 0.37; *P* = 0.001). Thus, although QTLs for traits that underpin reproductive isolation are scattered across the genome, there is nonetheless significant clustering among traits. Inversions can be important for maintaining linkage disequilibria between traits that confer reproductive isolation as they suppress recombination^34^. However, with the exception of chromosome 15, in which a known inversion is associated with colour pattern differences between *H. elevatus* and *H. pardalinus*^35^, we found no candidate inversions overlapping QTL peaks (Extended Data Fig. 7).

## Speciation was driven by introgression

The question of how new species originate and adapt to environments is fundamental to evolutionary biology. Hybridization might have a key role in establishing barriers to gene flow by creating new allelic combinations^36,37^. Many genomic studies have provided evidence of admixture among species^4,38–41^, but convincing cases of homoploid hybrid speciation remain scarce^1,3,6^. Here we show that *H. elevatus* is a hybrid species, the origin of which was triggered by introgression of traits from *H. melpomene* into a *H. pardalinus*-like ancestor (Fig. 1a). These traits place *Heliconius elevatus* on a separate adaptive peak and allow it to coexist in sympatry with both parental species, despite occasional but pervasive gene flow with *H. pardalinus* that distorts the evolutionary history of 99% of the genome away from the species tree. Furthermore, we estimate that *H. elevatus* has persisted in widespread sympatry as a distinct lineage for over 720,000 generations, suggesting that it is stable and not in the process of fusing with *H. pardalinus*. To our knowledge, this makes it the oldest reported case of homoploid hybrid speciation, and our study is among the few to fulfil the strict criteria for hybrid speciation that were laid out in a previous study^3^. Because *H. elevatus* overlaps broadly across its Amazonian distribution with both progenitors, it also differs from most other previously described putative hybrid species^42–44^, including *Heliconius heurippa*^45^, which overlap with only one or neither of the parental lineages. Consistent with models of sympatric speciation^46^, traits conferring mate choice and divergent selection are clustered within the genome. Nonetheless, there are multiple clusters of these species-specific QTLs across different chromosomes. Adaptive coupling among these unlinked loci therefore spreads the effects of selection across the genome, allowing multiple genomic regions to evolve as a coadapted unit^47–51^. The capacity of this multilocus genetic architecture to resist gene flow indicates that sympatric speciation can occur more readily than predicted by simple theory based on small numbers of traits or loci.

## Methods

### Data collection and whole-genome resequencing

#### Collections and library preparation

Adult butterflies were collected between 2009 and 2018 and stored at −20 °C in either salt-saturated dimethyl sulfoxide or 100% ethanol. RNA-free genomic DNA was extracted from the thorax of butterflies using Qiagen Blood and Tissue and E.Z.N.A Tissue DNA kits (Omega Bio-tek), and used to prepare 350-bp insert Illumina libraries for 33 individuals, which were sequenced using 100–150-bp paired-end sequencing on Illumina instruments. Collecting and export permit numbers are provided in the Acknowledgements. We complemented these samples with previously published sequences (see Supplementary Table 1 for sample details).

#### Read filtering, mapping and genotype calling

After demultiplexing, reads were filtered for Illumina adapters using cutadapt v.1.8.1 (ref. ^52^) and then mapped to the *H. melpomene* assembly v.2.5 (Hmel2.5, ref. ^53^)(ref. ^54^) using BWA-MEM v.0.7.15 (ref. ^55^) with default parameters and marking short split hits as secondary. Mapped reads were sorted and duplicate reads removed using sambamba v.0.6.8 (ref. ^56^) sort and markdup modules, respectively. Mapped reads were further realigned around indels using the Genome Analysis Toolkit (GATK) v.3.8 RealignerTargetCreator and IndelRealigner modules^57,58^, to reduce the number of indel miscalls. Read depth and other relevant read alignment quality control metrics were computed using QualiMap v.2.2.1 (ref. ^59^).

Genotype calling was performed using the bcftools v.1.5 (ref. ^60^) mpileup and call modules, requiring a minimum MQ (mapping quality) and QUAL (base quality) of 20. Genotyping was performed jointly for individuals belonging to the same population using the multiallelic and rare-variant calling option (-m) in bcftools call. Ploidy aware genotype calling was performed for the Z chromosome. Genotypes were filtered using the bcftools filter module. Both invariant and variant sites were required to have QUAL (quality of the variant call) ≥ 20 and MQ (root mean square mapping quality) ≥ 20, with DP (read depth) ≥ 8 for individual genotypes (DP ≥ 4 for females on the Z chromosome) and GQ (genotype quality) ≥ 20. All genotypes not fulfilling these criteria or within 5 bp of an indel (--SnpGap) were recoded as missing data.

### Species relationships and demographic modelling of hybrid speciation

Relationships between *H. elevatus*, *H. pardalinus*, *H. melpomene* and other closely related species were investigated by building a phylogenetic network. The dataset was filtered to include only biallelic sites (excluding singletons) without missing data and at least 1 kb apart, using Plink v.1.9 (ref. ^61^). Pairwise absolute genetic distances between all pairs of samples were calculated using the disMat.py script (https://github.com/simonhmartin/genomics_general). The distance matrix was then used to construct a phylogenetic network using the NeighbourNet approach^62^, implemented in SplitsTree v.4.15.1 (ref. ^63^), with default parameters.

We also investigated species relationships by estimating a concatenated neighbour-joining tree. In this analysis, we included both variable and invariable sites, at least 1 kb apart and without missing data. The neighbour-joining tree was estimated from individuals’ pairwise distances using the R package ape v.5.7 (ref. ^64^) ‘read.dna’ and ‘nj’ functions. Trees were rooted using the ‘midpoint’ function from the R package phangorn v.2.11.1 (ref. ^65^). Bootstrap supports were obtained on the basis of 100 bootstrap replicates, using the ‘boot.phylo’ function in the R package ape v.5.7 (ref. ^64^).

Genealogical relationships along the genome between the three focal species (*H. elevatus*, *H. pardalinus* and *H. melpomene*) were further investigated using TWISST^66^ (https://github.com/simonhmartin/twisst), and using *Heliconius nattereri* as an outgroup species. Only SNPs fixed in the outgroup (*H. nattereri*), variable in the focal species and with a minimum allele frequency (MAF) of 0.05 were considered. Statistical phasing and imputation were performed using Beagle v.5.1 (ref. ^67^), with default settings. The phased filtered dataset was used to infer neighbour-joining phylogenies for non-overlapping windows of 1,000 SNPs (median size of around 23.6 kb), assuming the GTR substitution model, in PHYML (ref. ^68^). Exact weightings were computed for all phylogenies. Windows were classified into each of the following categories when weighting support was 0.5 or greater: (i) *H. elevatus* and *H. pardalinus* group together but are not reciprocally monophyletic; (ii) *H. elevatus* and *H. pardalinus* group together and are reciprocally monophyletic; (iii) *H. elevatus* and *H. melpomene* group together but are not reciprocally monophyletic; and (iv) *H. elevatus* and *H. melpomene* group together and are reciprocally monophyletic.

To infer the timing of introgression from *H. melpomene* into *H. elevatus* and its split from *H. pardalinus*, we used the multispecies coalescent-with-introgression (MSCi) model implemented in BPP v.4.6.2 (ref. ^22^) (A00 analysis). For each species of the three species, we selected four individuals to generate sequenced alignments. For *H. melpomene*, we used *H. melpomene aglaope* from Peru. Given the population structure between Amazonian and non-Amazonian population of both *H. elevatus* and *H. pardalinus* and evidence for gene flow between the two species in the Amazon, we first performed this analysis using the non-Amazonian populations (that is, *H. elevatus bari* and *H. pardalinus sergestus*). Loci were selected randomly from autosomes, requiring loci to be 2 kb long, a minimum distance of 20 kb to the next closest locus and 5 kb from the closest exon as annotated in *H. melpomene* assembly v.2.5. For each locus, individuals with more than 20% missing data and sites containing missing genotype calls were removed. Only loci containing all individuals and 800 bp passing filters were considered. Heterozygous sites were assigned IUPAC ambiguity codes. Demographic parameter estimation was performed using a fixed species tree, with introgression events (see Fig. 1e and Extended Data Fig. 1). An inverse gamma prior (invG) was applied both to the root age (*τ*_0_) and to all populations’ effective population sizes (*θ*) – invG(*a* = 3, *b* = 0.06) and invG(*a* = 3, *b* = 0.04), respectively. A beta prior was applied to the introgression probability (*j*) – Beta(*a* = 1, *b* = 1). The MCMC was run for 1,000,000 iterations after 50,000 iterations of burn-in, sampling every 10 iterations.

### Historic and recent gene flow

#### Species-diagnostic SNPs

To characterize instances of recent gene flow between Amazonian *H. pardalinus* and *H. elevatus*, we relied on ancestry-informative SNPs (allele frequency difference ≥ 0.8) between these two groups. Only ancestry-informative SNPs at least 10 kb apart were considered. For each SNP, an ancestry score of 0 and 1 was assigned for *H. elevatus* homozygous and *H. pardalinus* homozygous variants, respectively, and 0.5 for heterozygous. We then calculate each individual’s ancestry (average ancestry across SNPs) and heterozygosity, on the basis of the ancestry-informative SNPs passing the filters. A custom R script was used to visualize genotypes of species-diagnostic SNPs across the genomes of different individuals. The same approach was used to determine species-diagnostic SNPs between Amazonian *H. elevatus* and *H. melpomene*.

#### *f*_4_ statistics

We calculated the *f*_4_ statistics in ADMIXTOOLS (ref. ^69^) to measure shared drift between pairs of populations of different species in the same location versus between pairs of populations of the same species in different locations. Shared drift between populations of different species in the same location is indicative of gene flow between species, and shared drift between populations of the same species in different locations is indicative of grouping by species. Only autosomal biallelic SNPs were considered in this analysis. Standard errors were estimated through a weighted block jackknife approach over 500-kb blocks. We also measured the Euclidean geographic distance between all possible pairs of locations and performed a Mantel test for its correlation with the *f*_4_ statistics.

#### Estimates of gene flow between population pairs

We used G-PhoCS (ref. ^70^) to estimate divergence times, effective population sizes and migration rates between pairs of populations of *H. elevatus* and *H. pardalinus*, both within and between species. In all analyses, we also include one individual from an outgroup species (*Heliconius besckei*) and estimate model parameters assuming possible bidirectional migration between the two ingroup species. G-PhoCS uses multiple independent neutrally evolving loci to infer demographic parameters. Therefore, we first defined regions of the genome within scaffolds larger than 1 Mb and at least 1 kb away from exons as annotated in *H. melpomene* assembly v.2.5. Within these regions we then selected 1-kb blocks that were at least 10 kb apart from the nearest block and produced sequence alignments, masking annotated repetitive elements and CpG islands identified with the software gCluster (ref. ^71^). Because previous studies have reported extensive introgression between both *H. elevatus* and *H. pardalinus* with other *Heliconius* species in large regions of the genome surrounding the three major colour pattern loci, we excluded blocks in chromosomes containing these loci (chromosomes 10, 15 and 18). We also excluded blocks in the Z chromosome owing to its different effective population size. For each alignment, we excluded individuals with more than 60% missing genotype calls, and only alignments with at least three individuals per population (or all individuals in the populations for those with fewer than three individuals) and a minimum of 100 bp for which no more than 25% of individuals had missing genotype calls were considered. We coded heterozygous genotype calls using IUPAC codes. A gamma prior with *α* = 2 and *β* = 100 was used for both the mutational-scaled effective population size (*θ*) and the divergence time (*τ*) between the two ingroup populations, whereas a gamma prior with *α* = 2 and *β* = 50 was used for the divergence time to the outgroup. For the mutation-scaled migration rates, we defined a gamma prior with *α* = 0.005 and *β* = 0.00001. The model was run three times, with a burn-in of 50,000 iterations (allowing for automatic fine-tuning of the parameters) followed by 200,000 iterations, sampling every 200 iterations. Convergence of the Markov chain and between the three different replicates was inspected using custom scripts. To convert the *θ* and *τ* estimates to absolute effective population size and divergence time, we assumed an average mutation rate (*µ*) of 2.9 × 10^−9^ substitutions per site per generation and an average generation time (*g*) of 0.25 years (ref. ^72^). We also obtain estimates of the effective migration rate (*N*_e_*m*) using the formula: *N*_e_*m*_AB_ = *M*_AB_ × *θ*_B_/4.

#### Simulations to infer robustness of G-PhoCS inferences

Whenever *Nm* > 1, estimates of *Nm* for the same population comparisons varied both in value and directionality between different replicate runs of G-PhoCS. To investigate the cause for these differences, we performed coalescent simulations using MSMS (ref. ^73^). We considered the same demographic scenario as for the G-PhoCS runs; that is, two sister populations (A and B) that diverged at *T*_D1_ and split from the outgroup (C) at *T*_D2_, and allowing either unidirectional or bidirectional migration between A and B. The split time between the two sister populations (*T*_D1_) was set to four million generations, and eight million generations for the split of the outgroup (*T*_D2_). An effective population size (*N*_e_) of one million or five million was assumed for the two ingroup populations (400,000 for the outgroup), and varying levels of gene flow (*Nm*) were considered (0.01, 0.1, 1.0, 2.0 and 10.0). For each scenario, we simulated 100 trees in MSMS (ref. ^73^), from which we generated sequence alignments using Seq-Gen v.1.3.4 (ref. ^74^). Custom scripts were used to combine pairs of haploid sequences into diploid sequences, using IUPAC codes for heterozygous sites, and to convert the alignments to the G-PhoCS sequence format. Finally, we ran G-PhoCS for the simulated datasets using the same settings as described above. Whenever *Nm* > 1 in the simulated datasets, G-PhoCS showed a similar behaviour to what was seen in our analysis of the *Heliconius* data (Supplementary Table 6). We believe that this effect is due to the difficulty of estimating gene flow when the populations are nearly panmictic. Hence, for each population pairwise comparison, the highest *Nm* estimate among the three replicate runs is presented in Fig. 2b.

#### Species-tree inference

Phylogenetic relationships between the *H. pardalinus* and *H. elevatus* major groups were inferred using the multispecies coalescent (MSC) approach implemented in BPP v.4.6.2 (ref. ^22^), while accounting for incomplete lineage sorting. Three *H. p. sergestus* individuals (with the highest coverage) and three *H. elevatus* individuals from the Guianas (the individual with the highest coverage per location (French Guiana, Suriname and Venezuela)) were considered. For Amazonian *H. pardalinus* and *H. elevatus*, again, only the individual with the highest coverage from each of three locations—Ecuador, Bolivia and Brazil—was included. For this analysis, loci were selected by first defining regions of the genome within scaffolds larger than 1 Mb. To minimize the effect of linked selection, these regions also had to be at least 2 kb from exons as annotated in *Heliconius melpomene* v.2.5 (Hmel2.5, ref. ^54^). Because the analysis assumes no intra-locus recombination and independence between loci, we selected loci of 100–250 bp and at least 2 kb from neighbouring loci. Sequence alignments were produced for all loci, masking repetitive elements as annotated in the reference genome and CpG islands identified with the software gCluster (ref. ^75^). For each locus, individuals with more than 50% missing genotype calls were excluded from the alignment and only loci with at least two individuals per population were considered. Furthermore, sites with more than 20% of individuals with missing genotype calls were removed and loci with less than 50 bp passing filters were excluded. Loci were grouped into blocks of 100 loci, and those overlapping the inversion on chromosome 15 were grouped in a separate block. Species-tree estimation was then performed in BPP v.4.6.2 using the A01 analysis (species-tree inference assuming no gene flow). Inverse gamma priors (invGs) were applied both to the root age (*τ*_0_) and to effective population sizes (*θ*) – invG(3, 0.06) and invG(3, 0.04), respectively. Parameters were scaled assuming a mutation rate of 2.9 × 10^−9^ substitutions per site per generation and a generation time of 0.25 years (ref. ^54^). The MCMC was run for 1,000,000 iterations after 50,000 iterations of burn-in, sampling every 10 iterations. Three independent runs were performed for each block, using different starting species trees, and only blocks showing consistency among the three independent runs were considered. The most abundant estimated tree across the genome showed both species to be paraphyletic with respect to each other (Extended Data Fig. 2). We believe that this non-taxonomic arrangement is due to gene flow, which is not accounted for in the model.

#### Demographic modelling by analysis of site-frequency spectra

To understand the prevalence of gene flow at different stages of the speciation history of *H. elevatus* and *H. pardalinus*, we performed demographic modelling based on analysis of the site-frequency spectrum (SFS) using fastsimcoal2 v.2.7.0.2 (ref. ^76^). For this analysis, we considered all Amazonian and non-Amazonian populations of *H. elevatus* and *H. pardalinus*. Individuals with more than 50% missing data were excluded from the analysis and only sites genotyped in at least 80% of the individuals (including all four *H. p. sergestus*) were considered. Furthermore, only sites at least 2 kb apart and at least 10 kb from exons were considered, to mitigate the effects of linkage disequilibrium and linked selection, respectively. We further excluded sites within repetitive regions as annotated in the *H. melpomene* assembly Hmel2.5. The 209,115 sites that were retained after filtering were polarized by assessing the allele present in three outgroup species—*H. besckei*, *Heliconius ismenius telchinia* and *Heliconius numata robigus*. From each of the outgroup species, we chose one individual with the highest coverage and assigned the ancestral allele to each site if it was genotyped and monomorphic in the outgroup species. The unfolded multidimensional site-frequency spectrum (multiSFS) was generated using easySFS (https://github.com/isaacovercast/easySFS), using the recommended down projection approach (four individuals of *H. p. sergestus*; 10 northeastern group *H. elevatus*; and 20 *H. pardalinus* and 20 *H. elevatus* individuals from the Amazon) to maximize the number of segregating sites while accounting for missing data. For each demographic model, fitting of the simulated multidimensional site-frequency spectra to the empirical data was maximized using the composite-likelihood method implemented in fastsimcoal v.2.7 (ref. ^77^). For all model parameters, we used wide search ranges from which initial starting parameter values were randomly sampled. For each model, we performed 100 independent fastsimcoal2 runs. Parameter estimates optimization was performed for 40 expectation-maximization cycles and the expected SFS was estimated using 100,000 coalescent simulations. The best fitting model was identified by means of the Akaike information criterion, considering for each model the optimization run with the highest likelihood (using the script https://github.com/speciationgenomics/scripts/blob/master/calculateAIC.sh). To account for stochasticity in the likelihood approximation, we further compared likelihood distributions of the different models by performing 100 independent runs from parameter values estimated under the most likely replicate run for each model. Finally, for the best fit model, confidence intervals around the maximum likelihood parameter estimates were obtained by nonparametric block-bootstrapping. For this, the 209,115 sites were divided into 100 blocks and sampled with replacement.

### Genomic islands of divergence and introgression

#### Summary statistics

We calculated between-population differentiation (*F*_ST_) for Amazonian and non-Amazonian populations of both *H. elevatus* and *H. pardalinus* groups, in sliding windows of 25 kb (5 kb step size) along the genome using the script popgenWindows.py (https://github.com/simonhmartin/genomics_general). The script implements a version of Hudson’s *K*_ST_ (ref. ^78^), modified to avoid weighting nucleotide diversity in each population by sample size. Individuals with more than 50% missing data were removed. Only sites with a maximum of two alleles, and with at least three individuals with genotype calls per population (or the total number of individuals in populations with fewer than three individuals) were considered. Only windows with at least 10% of sites passing filters were considered in the analysis.

#### Topology weighting

To determine genomic regions in which *H. elevatus* and *H. pardalinus* are reciprocally monophyletic (that is, genomic regions that are potentially involved in species barriers), genealogical relationships between Amazonian and non-Amazonian populations along the genome were quantified using TWISST^66^ (https://github.com/simonhmartin/twisst). The same dataset as for *F*_ST_ was used, but also adding five individuals of representative outgroup species (*H. besckei*, *H. ismenius*, *H. numata*, *H. nattereri* and *H. ethilla*). Statistical phasing and imputation were performed using Beagle 5.1 (ref. ^67^), with default settings. Only SNPs fixed in all outgroup individuals and variable in the ingroup population with an MAF of 0.05 were considered. The phased filtered dataset was used to infer neighbour-joining phylogenies for windows of 100 SNPs (slide every 25 SNPs), assuming the GTR substitution model, in PHYML (ref. ^68^). Exact weightings were computed for all phylogenies. To estimate the proportion of trees supporting a grouping of individuals by species versus grouping by geography, we considered five groups: (i) *H. elevatus* from the Guianas (Venezuela, Suriname and French Guiana); (ii) *H. elevatus* from the Amazon; (iii) *H. pardalinus* from the Amazon; (iv) *H. p. sergestus* (Andes); and (v) an outgroup, *H. nattereri*. Because we hypothesize that introgression from *H. melpomene* into *H. elevatus* could be involved in speciation of the latter and *H. pardalinus*, the same analysis was performed including only Amazonian *H. elevatus*, Amazonian *H. pardalinus* and two *H. melpomene* populations (*H. m. amaryllis* and *H. m. aglaope*). By including *H. ethilla* (a sister species to *H. elevatus* and *H. pardalinus*) as a fifth population, we were able to polarize the genealogies, allowing determination of the direction of introgression.

#### Association between *H. melpomene* introgression and genomic islands of divergence

To test whether *H. melpomene* introgression in the genome of *H. elevatus* is associated with genomic islands of divergence between sympatric *H. elevatus* and *H. pardalinus*, we performed a Fisher’s exact test. First, we defined genomic islands of divergence as regions with *F*_ST_ ≥ 0.2 and in which TWISST recovered both *H. pardalinus* and *H. elevatus* as reciprocally monophyletic (with weight ≥ 0.8). Second, we defined as introgressed, genomic regions in which TWISST grouped *H. elevatus* with *H. melpomene* with a weight ≥ 0.8. We then performed a Fisher’s exact test, as implemented in bedtools v.2.30.0 (ref. ^79^), to test whether the two sets of genomic intervals overlap more than expected given the size of the reference genome.

### Genetic mapping of traits involved in reproductive isolation

Captive populations of Amazonian *H. elevatus pseudocupidineus*, *H. pardalinus butleri* and *H. m. agalope* were established in outdoor insectaries in Tarapoto, Peru and in heated indoor insectaries in York, UK, as previously described^17^. Crosses for QTL mapping were generated by mating *H. elevatus* with *H. pardalinus* to produce F_1_ broods, and then by either crossing these amongst themselves to generate F_2_ broods or backcrossing to parental taxa.

#### Colour pattern phenotyping

Dorsal surfaces of wings from 12 *H. elevatus*, 19 *H. pardalinus*, 14 *H. m. aglaope*, 348 F_2_ and 50 backcross hybrids were photographed in a light box against a white background using a Canon EOS D1000 together with an X-rite ColorChecker Mini (Supplementary Table 7). From each image, we selected a single forewing and hindwing for analysis, clipped the image to the wing outlines and flipped wings when necessary to ensure that all were similarly orientated (resulting in two files; one forewing and one hindwing). To align the wings so that pixels represent homologous units among individuals, we used image registration^80^, a regression-based method that aligns two sets of wings (a source and a reference) according to intensity-based similarity. We chose the reference set of wings using the PCA of wing shape (see below). For forewing (36 PCs) and hindwing (26 PCs) we found the mean value for each PC across all F_2_ and backcross individuals. We assigned the reference individual as the individual that had the minimum deviation from these mean values (summed across all PCs). We then checked all alignments by eye. To allow for minor misalignment or damage to wings, we included pixels in which up to 5% of individuals had missing RGB values.

#### Wing shape

Wing shape was quantified in 31 *H. elevatus*, 26 *H. pardalinus*, 10 *H. m. aglaope* and 308 F_2_ and 36 backcross hybrids using landmark-based geometric morphometrics analyses (Supplementary Table 7). The ventral side of the butterfly wings was scanned using a flatbed scanner at 300 dpi and landmarks were placed at specific vein intersections^81^ on the forewing (20 landmarks) and hindwing (15 landmarks) using tpsDig2^82^. Landmark coordinates were adjusted for size and orientation using a Procrustes analysis from the package geomorph^83^. Forewings and hindwings were analysed separately.

#### Flight

*H. elevatus* (*n* = 12), *H. pardalinus* (*n* = 13), *H. m. aglaope* (*n* = 5) and F_2_s (*n* = 40) were filmed flying freely in a large flight cage (5 × 2.5 × 2 m) using a GoPro HERO 4 Black camera at 239.7 frames per second at a resolution of 720p (Supplementary Table 7). Videos were studied in slow motion using GoPro Studio v.2.5.9.2658. Flight sequences in which an individual was flying straight and level for at least five wing beats were selected to measure wing beat frequency (WBF). WBF was measured by counting the number of complete wing beats and the number of video frames. Five WBF measurements were taken per individual from separate flight sequences and used to calculate the individuals’ mean WBF by dividing the total number of wing beats across all flight sequences by the total flight time estimated from the number of video frames.

#### Female host plant preference

Host plant preference assays for QTL mapping were performed by introducing single *H. elevatus*, *H. pardalinus* and F_2_ females (*n* = 24, 32 and 31, respectively) into cages measuring 1 m (*W*) × 2 m (*L*) × 1.7 m (*H*), with two approximately equally sized shoots of the host plants (*P. riparia* and *P. venusta*) placed in the back corners. At the end of each day, the number of eggs laid on each plant species was recorded and the eggs were removed (Supplementary Table 7). To compare the oviposition preference of Peruvian *H. elevatus*, *H. pardalinus* and *H. melpomene*, groups of females (wild-caught and/or reared) of a given taxon were released into a large cage (2.5 m (*W*) × 5 m (*L*) × 2 m (*H*)) containing single representatives of 21 species of *Passiflora* that are commonly found near Tarapoto, Peru and which represent potential host plants^17^. The number of eggs laid on each host plant was recorded at the end of each day. A total of 126 females were tested, resulting in a total of 889 eggs (176 from 35 *H. elevatus* females, 288 from 24 *H. melpomene* and 425 from 51 *H. pardalinus*).

#### Male sexual preference

To assay male preference for female colour pattern, we presented *H. elevatus*, *H. pardalinus* and F_2_ males (*n* = 46, 66 and 106, respectively) with a pair of model female wings (one *H. elevatus* and one *H. pardalinus*), and recorded courtship events (full details of the experimental set-up are provided in ref. ^17^). Males were tested individually and placed in the experimental cage one day earlier to allow acclimatization. Trials lasted 15–30 min. The number of courtships (defined as sustained flight 5–15 cm over a model) by the males directed towards each of the model wings was recorded (Supplementary Table 7).

#### Phenotyping of androconial volatiles

Male *Heliconius* produce complex chemical blends of volatile compounds from their hindwing androconia. These blends have been shown to function as sex pheromones in several other *Heliconius* species and in butterflies in general^84,85^. Androconial regions were excised from 13 *H. elevatus*, 10 *H. pardalinus*, 7 *H. melpomene malleti* individuals and 122 F_2_ and 17 backcross hybrids 21 days after eclosion, and suspended in dichloromethane. The extracts were analysed by gas chromatography–mass spectrometry (GC–MS), as reported previously^16,86^ (Supplementary Table 7) on a 7890A GC-System coupled with an MSD 5975C mass analyser (Agilent Technologies) instrument fitted with an HP-5MS column (50 m, 0.25 mm internal diameter, 0.25 µm film thickness). The ionization method was electron impact with a collision energy of 70 eV. Conditions were as follows: inlet pressure 9.79 psi, He 20 ml min^−1^, injection volume 1 µl. The GC was programmed as follows: start at 50 °C, increase at 5 °C min^−1^ to 320 °C and hold that temperature for 5 min. The carrier gas was He at 1.2 ml min^−1^. For all identified compounds, the concentration was calculated from the peak’s area, as reported by AMDIS software^87^. Each compound’s chromatogram was interpreted by AMDIS through the NIST databases and the additional databases compiled at the Institute of Organic Chemistry of Technische Universität Braunschweig. All identifiable compounds running between undecane and nonacosanal were scored. Potential contaminants or extraneous compounds were excluded, together with compounds that appeared fewer than 10 times across the entire dataset.

#### DNA extraction and RAD library preparation for QTL analysis

RNA-free genomic DNA was extracted from thoracic tissue using a Qiagen DNeasy Blood and Tissue Kit following manufacturer’s standard protocol. Restriction-site-associated DNA (RAD) libraries were prepared using a protocol modified from (ref. ^88^, using a *PstI* restriction enzyme, sixteen 6-bp P1 barcodes and eight indexes. DNA was Covaris sheared to 300–700 bp and gel size selected. A total of 128 individuals were sequenced per lane, with 125-bp paired-end reads, on an Illumina HiSeq 2500 (Supplementary Table 8).

#### SNP calling

Fastq files from each RAD library were demultiplexed using process_radtags from Stacks^89^, and BWA-MEM^90^ was used with default parameters to map the reads to the *H. melpomene* assembly v.2.5 (ref. ^91^). BAM files were then sorted and indexed with Samtools (ref. ^90^), and Picard v.1.119 (https://github.com/broadinstitute/picard) was used to add read group data and mark PCR duplicates. To check for errors, confirm pedigrees and assign samples with unrecorded pedigree to families, we used Plink v.1.9 (ref. ^61^) to estimate the fraction of the genome that is identical by descent (IBD; $\widehat{{\boldsymbol{\pi }}}$) between all pairwise combinations of samples (siblings and parent-offspring comparisons should yield $\widehat{{\boldsymbol{\pi }}}$ values close to 0.5). In addition, for specimens that were sequenced multiple times, we checked that samples derived from the same individual $(\widehat{{\boldsymbol{\pi }}}\approx 1)$. We then merged these samples, using the MergeSamFiles command from Picard Tools, and used Samtools mpileup command to call SNPs.

#### Linkage map construction

Linkage maps were built for hybrid and within-species crosses using Lep-MAP3 (ref. ^92^). Pedigrees are provided in Supplementary Table 8. SNPs were first converted to posterior genotype likelihoods for each of ten possible SNP genotypes. We used the ParentCall2 module to correct erroneous or missing parental genotypes and call sex-linked markers using a log-odds difference of >2 (ZLimit) and halfSibs = 1. We used Filtering2 to remove SNPs showing segregation distortion, specifying a *P* value limit of 0.01; that is, there is a 1:100 chance that a randomly segregating marker is discarded. We then separated markers into chromosomes using their Hmel2.5 scaffold. To obtain genetic distances between markers, we fixed the order of the markers to their order in Hmel2.5, and then evaluated this order, using all markers and specifying no recombination in females. We then used map2gentypes.awk to convert the Lep-MAP3 output to four-way fully informative genotypes with no missing data. To assign ancestry to phased haplotype blocks in the hybrid linkage map, we used biallelic sites with significantly different allele frequencies in the parental species (*χ*^2^ test applied to sequences for 26 *H. elevatus* and 47 *H. pardalinus* individuals from Peru and Ecuador).

#### QTL mapping

The colour pattern, androconial volatiles and wing shape datasets are multivariate and highly collinear. We therefore used PCA to reduce the phenotypic values for the hybrids to orthogonal vectors (PCs), which we then used as phenotypes in QTL mapping. For wing shape, we applied PCA to the Procrustes coordinates. For the androconial volatiles, we applied the PCA to the set of compounds that were significantly different between the two parental species (one-tailed paired *t*-test). For colour pattern, we performed a PCA on the concatenated RGB values from the aligned images and retained PCs that explained more than 1% of the variance.

For colour pattern, androconial volatiles, wing shape and WBF, we tested for associations between phenotype and genotype using linear models with normal errors. For wing shape, we included centroid size as a covariate to control for allometry. For female host plant choice and male preference for female colour pattern, we (i) logistically transformed the proportions and used linear models with normal errors; and (ii) used generalized linear mixed models with an individual-level random effect to account for overdispersion and binomial errors. The significance of QTL scans was assessed by permuting the phenotypes relative to the genotypes (1,000 permutations). For traits phenotyped in both males and females, a sex-specific significance threshold was used to avoid spurious sex linkage (see Supplementary Table 5).

We first analysed all data using F_2_s only, using R/qtl (ref. ^93^) to estimate genotype probabilities at 1-cM intervals, using the Haldane mapping function and an assumed genotyping error rate of 0.001. These genotype probabilities were then used as the dependent variable in models, and for traits phenotyped in both males and females we included sex and cross direction as covariates for markers on the sex chromosome. For traits for which backcrosses had been scored in addition to F_2_s, we performed an additional round of analyses combining F_2_s with backcrosses. In this case, we used the categorical genotypes (EE, EP and PP) inferred from linkage mapping as the dependent variables, and added random effects for cross type (three levels: F_2_, backcross to *H. elevatus*, backcross to *H. pardalinus*), sex or individual. Model structures and estimated coefficients are provided in Supplementary Table 5.

To test whether QTLs are significantly clustered (that is, genetically linked), for each QTL we estimated the recombination probability with its nearest neighbouring QTLs (using the position of the maximum LOD score), and took the mean of the resulting vector (low values indicate that most QTLs are linked to at least one other QTL; high values indicate that most QTLs are unlinked). We then randomized the position of the QTLs 10,000 times and compared the observed data to the randomized dataset using a two-tailed test (*P* = the proportion of randomized datasets that give a result more extreme than the observed data × 2). When multiple QTLs overlapped within the phenotypic classes forewing colour pattern, hindwing colour pattern, forewing shape and hindwing shape, we included only the best supported QTL (highest LOD score). To test whether species and introgression topologies are associated with QTLs, we applied the same test.

To identify putative structural rearrangements between *H. elevatus* and *H. pardalinus*, we compared recombination rates between F_2_s and within-species crosses (F_2_s, 441 individuals across 26 families; *H. elevatus*, 179 individuals across 9 families; *H. pardalinus*, 296 individuals across 15 families). Regions that are freely recombining within species but not in F_2_s represent candidate rearrangements that might facilitate divergence and speciation. The probability of the within-species recombination events observed within an F_2_ breakpoint can be given as *p*^*n*^, where *p* is the fraction of parental individuals in the mapping crosses and *n* is the observed number of recombination events. We estimated *p*^*n*^ within each F_2_ breakpoint and considered breakpoints in which *p* < 0.01 to be candidate rearrangements.

### Reporting summary

Further information on research design is available in the Nature Portfolio Reporting Summary linked to this article.

## Online content

Any methods, additional references, Nature Portfolio reporting summaries, source data, extended data, supplementary information, acknowledgements, peer review information; details of author contributions and competing interests; and statements of data and code availability are available at 10.1038/s41586-024-07263-w.

## Supplementary information

Supplementary Information A full guide for Supplementary Tables 1–8 (Tables supplied separately).

Reporting Summary

Supplementary Tables Supplementary Tables 1–8.

## Data Availability

Newly generated whole-genome sequencing data used in the population genomic analyses and RAD-sequencing data used in the cross analyses have been uploaded to the NCBI Sequence Read Archive (SRA) (PRJNA1074694). NCBI SRA accessions for individual samples are listed in Supplementary Tables 1 and 8. Phenotypic data are available in Supplementary Table 7 and at 10.5281/zenodo.10685466 (ref. ^94^) and 10.5281/zenodo.10689714 (ref. ^95^).

## Code availability

Custom code used for the genomic analyses (https://github.com/FernandoSeixas/HeliconiusHybridSpeciation) and the QTL mapping (https://github.com/heliconius-maps/HeliconiusHybridSpeciation) is available from GitHub.
