# Supplementary material for: Hybrid speciation driven by multilocus introgression of ecological traits
Source: Nature. 2024 Apr 17;628(8009):811–7. doi: 10.1038/s41586-024-07263-w (PMC11041799; doi:10.1038/s41586-024-07263-w)
Supplement: Supplementary file 1 — A full guide for Supplementary Tables 1–8 (Tables supplied separately). [file 41586_2024_7263_MOESM1_ESM.pdf]

nature portfolio

https://doi.org/10.1038/s41586-024-07263-w

Supplementary information

Hybrid speciation driven by multilocus introgression of ecological traits

In the format provided by the authors and unedited

nature portfolio

https://doi.org/10.1038/s41586-024-07263-w

Supplementary information

Hybrid speciation driven by multilocus introgression of ecological traits

In the format provided by the authors and unedited

Supplementary Information Guide.

STable1.SamplingInfo. Detailed information of samples included in this study, for which whole genome sequencing data was available or generated. For each sample we provide the following descriptors (when the information is available): *Species/Subspecies*: Species and subspecies assignment based on phenotype. *Code*: original unique code assigned to the specimen. *Population Code:* unique population code specifying the population to which each specimen was assigned. *Country/Region/Province/Locality:* Geographic descriptors of the location from where the sample were collected. *Latitude/Longitude:* Geographic coordinates of the location from where the sample was collected. When exact coordinates were not available, approximate coordinates were chosen based on locality. *Molecular Sex:* individuals sex determined based on the median coverage of the Z-chromosome relative to median coverage of autosomes: ~0.5 for females (F) and ~1 for males (M). *Mean Coverage:* mean coverage calculated in 25-kb windows. *Sample Accession:* NCBI SRA BioSample accession identifiers. Samples sequenced in this study are highlighted in bold.

STable2.GPhoCS. Parameter estimates under the Isolation-with-Migration (IM) demographic model. Both raw values and scaled parameters (assuming a mutation rate of 2.9×10^{-9} mutations/site/generation) are presented. Population codes follow the notation of STable1.SamplingInfo.

STable3.fastsimcoal_Best_Model. Maximum-likelihood parameter estimates under the best fitting demographic model inferred based on the site-frequency (SFS) spectrum using fastsimcoal2 (see Extended Data Fig. 4). Median, minimum, maximum and 95% confidence interval estimates were obtained by nonparametric block-bootstrapping (see Methods). Parameters were scaled assuming a mutation rate of 2.9×10^{-9} mutations/site/generation and four generations per year. Abbreviations: Effective population sizes (N_i) of *H. pardalinus* Andes (N_1), *H. pardalinus* Amazon (N_2), *H. elevatus* Amazon (N_3), *H. elevatus* Guianas (N_4), common ancestor of *H. pardalinus* (N_{A1}), common ancestor of *H. elevatus* (N_{A2}); common ancestor of *H. pardalinus* and *H. elevatus* (N_{A3}). T_{SC}: Time of secondary contact between *H. pardalinus* and *H. elevatus* in the Amazon; T_{Div1}: time of split between *H. pardalinus* (Andes) and *H. pardalinus* (Amazon); T_{Div2}: time of split between *H. elevatus* (Guianas) and *H. elevatus* (Amazon), T_{Div3}: time of split between the common ancestor of *H. pardalinus* ($_{A1}$) and the common ancestor of *H. elevatus* ($_{A2}$). Effective migration rate (Nm_{xy}) from *x* to *y* (forward in time). Both the maximum estimated likelihood (MaxEstLhood) and maximum observed likelihood (MaxObsLhood) are provided.

STable4.f4tests. No evidence of gene flow between *H. elevatus* and *H. melpomene*, based on four population (f_4) tests. For each species, we selected populations from two different locations (*H. elevatus*: pop1 and pop2; *H. melpomene* (pop3 and pop4). The two locations are the same in both species: location X (pop1 and pop3) and location Y (pop2 and pop4). Gene flow between species within the same location is expected to result in significantly positive f_4 values. The *Pair Type* column indicates whether the selected locations are both in the Amazon basin (Amazon-Amazon) or in the Amazon basin and the Guianas (Amazon-Guianas). Abbreviations: *est*: estimated f_4 values; *se*: standard error; *z*: z-score; *p*: p-value.

STable5.QTLs. QTL positions, confidence intervals and details of models and coefficients. Cross F2 refers to analysis using F2 hybrids only, F2 + BC refers analysis of F2 and backcross hybrids together. Phenotype 1 gives the broad phenotypic class (colour pattern, wing shape, flight, host plant, pheromones or male preference), Phenotype 2 gives sub-categories of phenotypes within these classes (e.g. the principal components). LOD_max = the LOD score at the QTL peak. chrom_max = The chromosome number. physical_peak and physical_limits – the Hmel2.5 coordinates for the QTL peak and credible

intervals. cM_max and cM_limits – the QTL peak and credible intervals in genetic map units (centimorgans). OLS = ordinary least squares, LMM = linear mixed model, GLMM = generalized linear mixed model. Response = response variable and transformation, if applied. Genotypes are given as E (*H. elevatus* ancestry) or P (*H. pardalinus* ancestry). The first three columns EE, EP and PP correspond to genotypes for QTLs located on autosomes. The next four columns (Ew, PP, Pw and EP) correspond to genotypes for QTLs on the sex chromosome. Ew and Pw are male genotypes, PP and EP are female genotypes. Centroid_size – coefficient for centroid covariate when analysing wing shape. Significance of coefficients is given as follows: ^p<0.1, p<0.05, **p<0.01,***p<0.001. var_rf_CROSS = variance of random effect for cross type (when analysing F2s and backcrosses using LMM). var_rf_SEX = variance of random effect for sex (when analysing both sexes using LMM). var_rf_OBS variance of observation-level random effect (corresponding to individual when using GLMM to analyse courtship odds for male preference or female preference for alternative host plants). R^2 values are given for analyses of F2 crosses using OLS. n.perms = number of permuations. If perm.Xsp = TRUE, separate permutations were run for the sex chromosome and autosomes.

STable6.GPhoCS-Simulations. **Simulations to evaluate the performance of G-PhoCS inferring the directionality and amount of gene flow.** Divergence time (Td1, Td2) and effective population size (NeA, NeB, NeC, NeAB, NeABC) values are given in units of one million. The G-PhoCS inferences were run three times for each set of simulated parameters to evaluate consistency in the inference of the demographic parameters.

STable7.Phenotypes. Details of phenotypic data for hybrid crosses and parental species. ID is a unique identifier for each butterfly and can be cross-referenced with **STable8.RADseq.** Measurements are given for female preference for alternative host plants, male preference for alternative female colour patterns, wing beat frequency. GCMS trace files for male pheromones and the images used for colour pattern phenotyping and morphometrics are available on Zenodo using the stated DOIs.

STable8.RADseq. RAD-seq data for QTL mapping. Demultiplexed Fastq files and their NCBI SRA BioSample accession identifiers, together with pedigree for crosses.
