# Supplementary material for: Hybrid speciation driven by multilocus introgression of ecological traits
Source: Nature. 2024 Apr 17;628(8009):811–7. doi: 10.1038/s41586-024-07263-w (PMC11041799; doi:10.1038/s41586-024-07263-w)
Supplement: Supplementary file 2 — Reporting Summary [file 41586_2024_7263_MOESM2_ESM.pdf]

nature portfolio

Corresponding author(s): Neil Rosser & James Mallet

Last updated by author(s): 21/2/24

Reporting Summary

Nature Portfolio wishes to improve the reproducibility of the work that we publish. This form provides structure for consistency and transparency in reporting. For further information on Nature Portfolio policies, see our Editorial Policies and the Editorial Policy Checklist.

Statistics

For all statistical analyses, confirm that the following items are present in the figure legend, table legend, main text, or Methods section.

n/a	Confirmed	
☐	☒	The exact sample size (*n*) for each experimental group/condition, given as a discrete number and unit of measurement
☐	☒	A statement on whether measurements were taken from distinct samples or whether the same sample was measured repeatedly
☐	☒	The statistical test(s) used AND whether they are one- or two-sided<br>*Only common tests should be described solely by name; describe more complex techniques in the Methods section.*
☐	☒	A description of all covariates tested
☐	☒	A description of any assumptions or corrections, such as tests of normality and adjustment for multiple comparisons
☐	☒	A full description of the statistical parameters including central tendency (e.g. means) or other basic estimates (e.g. regression coefficient) AND variation (e.g. standard deviation) or associated estimates of uncertainty (e.g. confidence intervals)
☐	☒	For null hypothesis testing, the test statistic (e.g. *F*, *t*, *r*) with confidence intervals, effect sizes, degrees of freedom and *P* value noted<br>*Give P values as exact values whenever suitable.*
☐	☒	For Bayesian analysis, information on the choice of priors and Markov chain Monte Carlo settings
☒	☐	For hierarchical and complex designs, identification of the appropriate level for tests and full reporting of outcomes
☐	☒	Estimates of effect sizes (e.g. Cohen's *d*, Pearson's *r*), indicating how they were calculated

Our web collection on statistics for biologists contains articles on many of the points above.

Software and code

Policy information about availability of computer code

Data collection	cutadapt v1.8.1, BWA mem v0.7.15, sambamba v0.6.8, Genome Analysis Toolkit (GATK) v3.8, QualiMap v2.2.1, bcftools v1.5, AMDIS, Stacks, SAMtools, Picard-tools v 1.119, Plink 1.9, Lep-MAP3
Data analysis	SplitsTree version 4.15.1, disMat.py and popgenWindows.py scripts (github.com/simonhmartin/genomics_general), Twisst (github.com/simonhmartin/twisst), Beagle 5.1, PHYML, BPP v4.6.2, ADMIXTOOLS 2.0.0, G-PhoCS version 1.3, gCluster, msms v2.1.1, Seq-Gen v1.3.4, fastsimcoal2 (version 2.7.0.2), easySFS, , R version 4.1.3, R/QTL version 1.50,

For manuscripts utilizing custom algorithms or software that are central to the research but not yet described in published literature, software must be made available to editors and reviewers. We strongly encourage code deposition in a community repository (e.g. GitHub). See the Nature Portfolio guidelines for submitting code & software for further information.

Data

Policy information about availability of data

All manuscripts must include a data availability statement. This statement should provide the following information, where applicable:

- Accession codes, unique identifiers, or web links for publicly available datasets
- A description of any restrictions on data availability
- For clinical datasets or third party data, please ensure that the statement adheres to our policy

All data will be made freely available following acceptance of the manuscript.

Research involving human participants, their data, or biological material

Policy information about studies with human participants or human data. See also policy information about sex, gender (identity/presentation), and sexual orientation and race, ethnicity and racism.

Reporting on sex and gender	*Use the terms sex (biological attribute) and gender (shaped by social and cultural circumstances) carefully in order to avoid confusing both terms. Indicate if findings apply to only one sex or gender; describe whether sex and gender were considered in study design; whether sex and/or gender was determined based on self-reporting or assigned and methods used. Provide in the source data disaggregated sex and gender data, where this information has been collected, and if consent has been obtained for sharing of individual-level data; provide overall numbers in this Reporting Summary. Please state if this information has not been collected. Report sex- and gender-based analyses where performed, justify reasons for lack of sex- and gender-based analysis.*
Reporting on race, ethnicity, or other socially relevant groupings	*Please specify the socially constructed or socially relevant categorization variable(s) used in your manuscript and explain why they were used. Please note that such variables should not be used as proxies for other socially constructed/relevant variables (for example, race or ethnicity should not be used as a proxy for socioeconomic status). Provide clear definitions of the relevant terms used, how they were provided (by the participants/respondents, the researchers, or third parties), and the method(s) used to classify people into the different categories (e.g. self-report, census or administrative data, social media data, etc.) Please provide details about how you controlled for confounding variables in your analyses.*
Population characteristics	*Describe the covariate-relevant population characteristics of the human research participants (e.g. age, genotypic information, past and current diagnosis and treatment categories). If you filled out the behavioural & social sciences study design questions and have nothing to add here, write "See above."*
Recruitment	*Describe how participants were recruited. Outline any potential self-selection bias or other biases that may be present and how these are likely to impact results.*
Ethics oversight	*Identify the organization(s) that approved the study protocol.*

Note that full information on the approval of the study protocol must also be provided in the manuscript.

Field-specific reporting

Please select the one below that is the best fit for your research. If you are not sure, read the appropriate sections before making your selection.

☐ Life sciences ☐ Behavioural & social sciences ☒ Ecological, evolutionary & environmental sciences

For a reference copy of the document with all sections, see nature.com/documents/nr-reporting-summary-flat.pdf

Ecological, evolutionary & environmental sciences study design

All studies must disclose on these points even when the disclosure is negative.

Study description	We combine population genomic analysis with quantitative trait locus mapping of species-specific traits to analyse a new case of hybrid speciation in Heliconius butterflies.
Research sample	92 wild-caught individuals from three species (Heliconius elevatus, Heliconius pardalinus and Heliconius melpomene) spanning their collective geographic ranges. 944 sequenced and phenotyped butterflies (Heliconius elevatus, Heliconius pardalinus and interspecific hybrids) reared in insectaries (derived from a sympatric population in northern Peru).
Sampling strategy	Sample sizes were determined by based on preliminary experiments and the prior experiences of the investigators (e.g. Rosser et al. 2019, Evolution, 73(9)).
Data collection	Genomic samples from the wild caught individuals were obtained from field work and through our network of collaborators, all of who are named as author on the paper, or from previously published sequences. Crosses were performed in Peru in custom made insectaries, before being exported for genome sequencing. Phenotyping was carried out in Peru and/or the University of York and Technische Universität Braunschweig.
Timing and spatial scale	Wild-caught butterflies collected to found insectary populations and subsequent crossing experiments were collected 2014-16 in northern Peru.
Data exclusions	No data were excluded from analysis.
Reproducibility	When phenotyping individuals, wherever possible we carried out multiple independent experiments with different observers to confirm that our parameter estimates were reproducible (e.g. see male preference estimates in Rosser et al. 2019, Evolution, 73(9)).<br><br>Studies performing multiple QTL analyses on correlated phenotypes risk type 1 errors because of multiple tests, thereby reducing reproducibility. To avoid this, we reduced correlated response variables to orthogonal vectors, which we used as phenotypes in QTL mapping.

Randomization	The core tenet of QTL analysis is that it leverages recombination in F1 hybrids to average away population structure present in parental species. In F2s, genomic variation unlinked to causal variants is therefore randomised with respect to phenotype.
Blinding	When phenotyping hybrids for QTL analysis the observer is blind to the individual's genotype. Logistical constraints prevented blinding when phenotyping parental taxa (for example, when recording courtship behaviours of a butterfly). However, measurements were carried out by multiple observers (see section on Reproducibility), who recorded data as objectively as possible.

Did the study involve field work? ☒ Yes ☐ No

Field work, collection and transport

Field conditions	Custom built insectaries in northern Peru.
Location	Tarapoto, Department of San Martin, Peru.
Access & import/export	SERFOR, the Peruvian Ministry of Agriculture, and the Área de Conservación Regional Cordillera Escalera issued the following collecting permits for work in Peru (0289-2014-MINAGRI-DGFFS/DGEFFS, 020-014/GRSM/PEHCBM/DMA/ACR-CE, 040–2015/GRSM/PEHCBM/DMA/ACR-CE). The Ministerio del Ambiente and Museo Ecuatoriano de Ciencias Naturales issued collecting permits for work in Ecuador (005-IC-FAU-DNBAPVS/MA). Permits for Brazil were issued by ICMBio (#52562-3, #10438-1) and the Conselho Nacional de Desenvolvimento Científico e Tecnológico – CNPq (Expediente PR no. 01300.000477/2016-49, portaria no. 4.628). Field collections in Colombia were conducted under the permit no. 530 issued by the Autoridad Nacional de Licencias Ambientales of Colombia (ANLA).
Disturbance	The species involved are not rare and not of conservation concern. Collections were carried out next to existing trails, with negligible impact on the surrounding habitat, in collaboration and following guidance of local researchers (named as authors), and with appropriate permission from local communities and authorities.

Reporting for specific materials, systems and methods

We require information from authors about some types of materials, experimental systems and methods used in many studies. Here, indicate whether each material, system or method listed is relevant to your study. If you are not sure if a list item applies to your research, read the appropriate section before selecting a response.

Materials & experimental systems

n/a	Involved in the study	
☒	☐	Antibodies
☒	☐	Eukaryotic cell lines
☒	☐	Palaeontology and archaeology
☐	☒	Animals and other organisms
☒	☐	Clinical data
☒	☐	Dual use research of concern
☐	☒	Plants

Methods

n/a	Involved in the study	
☒	☐	ChIP-seq
☒	☐	Flow cytometry
☒	☐	MRI-based neuroimaging

Animals and other research organisms

Policy information about studies involving animals; ARRIVE guidelines recommended for reporting animal research, and Sex and Gender in Research

Laboratory animals	The study did not involve laboratory animals.
Wild animals	Wild butterflies were caught with entomological nets and transported in glassine envelopes. Individuals to be used for population genomic analyses were sacrificed the same day. Insectary reared individuals were sacrificed at varying ages depending on the phenotype being recorded (see Methods of main text). Butterflies were sacrificed using standard practice for Lepidoptera (a sharp pinch to the thorax).
Reporting on sex	Controlling for sex-linkage in QTL analyses requires careful consideration. Spurious sex-linkage can be produced depending on cross design and whether males and/or females are being analyses, meaning sex-specific permutation thresholds for statistical significance of QTLs may or may not be required. We followed Broman et. al (2006, Genetics 174, no. 4) when performing QTL analysis on sex chromosomes. Our choice of whether or not to specify sex-specific permutation thresholds for a given phenotype is recorded in the column perm.Xsp in Supplementary Table 5.
Field-collected samples	Butterfly crosses were performed in custom built insectaries, details can be found in Rosser et al. Evolution, 73(9).

Ethics oversight	Ethical guidance was provided by the Department of Biology Ethics Committee (University of York). As the research involved using small numbers of wild-caught individuals of an invertebrate species with negligible ecological impact, no specific ethical approval was required.

Note that full information on the approval of the study protocol must also be provided in the manuscript.

Dual use research of concern

Policy information about dual use research of concern

Hazards

Could the accidental, deliberate or reckless misuse of agents or technologies generated in the work, or the application of information presented in the manuscript, pose a threat to:

No	Yes	
☒	☐	Public health
☒	☐	National security
☒	☐	Crops and/or livestock
☒	☐	Ecosystems
☒	☐	Any other significant area

Experiments of concern

Does the work involve any of these experiments of concern:

No	Yes	
☒	☐	Demonstrate how to render a vaccine ineffective
☒	☐	Confer resistance to therapeutically useful antibiotics or antiviral agents
☒	☐	Enhance the virulence of a pathogen or render a nonpathogen virulent
☒	☐	Increase transmissibility of a pathogen
☒	☐	Alter the host range of a pathogen
☒	☐	Enable evasion of diagnostic/detection modalities
☒	☐	Enable the weaponization of a biological agent or toxin
☒	☐	Any other potentially harmful combination of experiments and agents
